# Histamine H_3_ receptor inverse agonists/antagonists influence intra-regional cortical activity and inter-regional synchronization during resting state: an exploratory cortex-wide imaging study in mice

**DOI:** 10.1186/s13041-024-01165-8

**Published:** 2024-11-27

**Authors:** Sentaro Kaita, Yoshikazu Morishita, Kenta Kobayashi, Hiroshi Nomura

**Affiliations:** 1 Endowed Department of Cognitive Function and Pathology, Institute of Brain Science, Nagoya City University Graduate School of Medical Sciences, 1 Kawasumi, Mizuho-cho, Mizuho-ku, Nagoya, 467-8601 Japan; 2 Section of Viral Vector Development, Center for Genetic Analysis of Behavior, National Institute for Physiological Sciences, Okazaki, 444-8585 Japan

**Keywords:** Histamine, H_3_ receptor, Somatosensory cortex, Synchronized activity, Spontaneous activity

## Abstract

The histaminergic system plays a key role in modulating learning and memory, wakefulness, and energy balance. Histamine H_3_ receptors constitutively inhibit the synthesis and release of histamine and other neurotransmitters. Therefore, H_3_ receptor inverse agonists/antagonists increase the synthesis and release of these neurotransmitters, enhancing cognitive functions, including memory consolidation and retrieval. Spontaneous neural activity across the cerebral cortex is essential for cognitive function, including memory consolidation. Abnormal spontaneous activity has, in fact, been associated with cognitive dysfunctions and psychiatric disorders. Given the cognitive improvement achieved with the use of H_3_ receptor inverse agonists/antagonists, we examined the effects of two inverse agonists/antagonists – thioperamide and pitolisant – on spontaneous cortical activity, using in vivo wide-field Ca^2+^ imaging. Changes in cortical activity, across multiple cortical regions and in inter-regional connectivity, from pre- to post-administration were evaluated using a linear support vector machine decoder. Thioperamide and pitolisant both modified the amplitude distribution of calcium events across multiple cortical regions, including a reduction in the frequency of low-amplitude calcium events in the somatosensory cortex. Graph theory analysis revealed increases in centrality measures in the somatosensory cortex with the use of both thioperamide and pitolisant, indicative of their importance in the organization of cortical networks. These findings indicate that H_3_ receptor inverse agonists/antagonists influence intra-regional cortical activity and inter-regional synchronization of activity in the cerebral cortex during the resting state.

**Supplementary Information:**

The online version contains supplementary material available at 10.1186/s13041-024-01165-8.

## Introduction

The histaminergic system of the brain originates from the tuberomammillary nucleus of the hypothalamus and projects widely throughout the brain [1–3]. This system modulates learning and memory, including the consolidation and retrieval of information, as well as wakefulness, motivation, and energy balance [4–7]. Activation of the histaminergic system promotes memory consolidation and retrieval, whereas its inhibition impairs these processes [8–12]. Histamine H_3_ receptors (H_3_R) are located on the soma and axon terminals of histaminergic neurons and constitutively inhibit the synthesis and release of histamine [13, 14]. These receptors also act as heteroreceptors, influencing the release of other neurotransmitters, such as acetylcholine, noradrenaline, dopamine, and GABA [15–18]. As such, H_3_R inverse agonist/antagonist drugs increase the synthesis and release of histamine and other neurotransmitters [19], enhancing memory retrieval and consolidation [7, 20, 21]. In a previous study, we demonstrated that H_3_R inverse agonists/antagonists promote histamine release, boosting perirhinal cortex (PRh) activity, resulting in restoration of retrieval of forgotten long-term object recognition memories in mice and facilitation of object memory retrieval in humans [22]. Therefore, H_3_R inverse agonists/antagonists may ameliorate cognitive impairments in various neuropsychiatric disorders [7].

Cortex-wide spontaneous neural activity is closely associated with cognitive functions. Resting-state activity in the primary visual and somatosensory cortices contributes to memory consolidation [23]. Furthermore, a functional magnetic resonance imaging (fMRI) study showed that resting-state activity across the entire cerebral cortex is similar to the activity patterns observed during various cognitive tasks, including sensory processing, behavioral control, and memory [24, 25]. Abnormal spontaneous activity has been associated with cognitive dysfunctions and psychiatric disorders. As an example, reduced functional connectivity within the frontoparietal control network, which includes regions such as the dorsolateral, posteromedial, lateral parietal, and posterior temporal cortices, has been identified in individuals with schizophrenia [26]. Similarly, in Alzheimer’s disease, there is a reduction in activity in the posterior cingulate cortex and hippocampus within the resting-state default mode network, with a decrease in the functional connectivity between these regions [27]. Furthermore, research in mice has demonstrated that optogenetic silencing of the spontaneous activity of auditory cortex neurons modulates memory formation and retrieval [28]. Taken together, these findings suggest that the spontaneous activity in the cerebral cortex is crucial for cognitive function.

Given that H_3_R inverse agonists/antagonists improve cognitive function, which is closely linked to cortical-wide spontaneous activity, we hypothesized that the inverse agonists/antagonists drugs would have an effect on cortical-wide spontaneous activity, modulating cognitive function. Histological studies have shown widespread projections of histaminergic neurons and expression of H_3_R throughout the cortex [1–3, 29, 30]. Physiological analyses focusing on individual cortical areas have revealed that histamine H_3_R inverse agonist/antagonist drugs increase c-Fos expression in the prefrontal and somatosensory cortices [31]. Additionally, in a previous study, we showed that pitolisant, an H_3_R inverse agonist/antagonist, increases synchronous activity and alters population activity in the PRh [32]. However, the effects of these inverse agonist/antagonist drugs on the widespread cortical activity and inter-regional networks, which are crucial for cognitive regulation, remain unclear. Accordingly, in this study, we used cortical-wide Ca^2+^ imaging [33] to examine how histamine H_3_R inverse agonists/antagonists influence spontaneous cortical activity in the resting state.

## Results

### Measured alterations in neuronal activity throughout the cerebral cortex

**Fig. 1:**
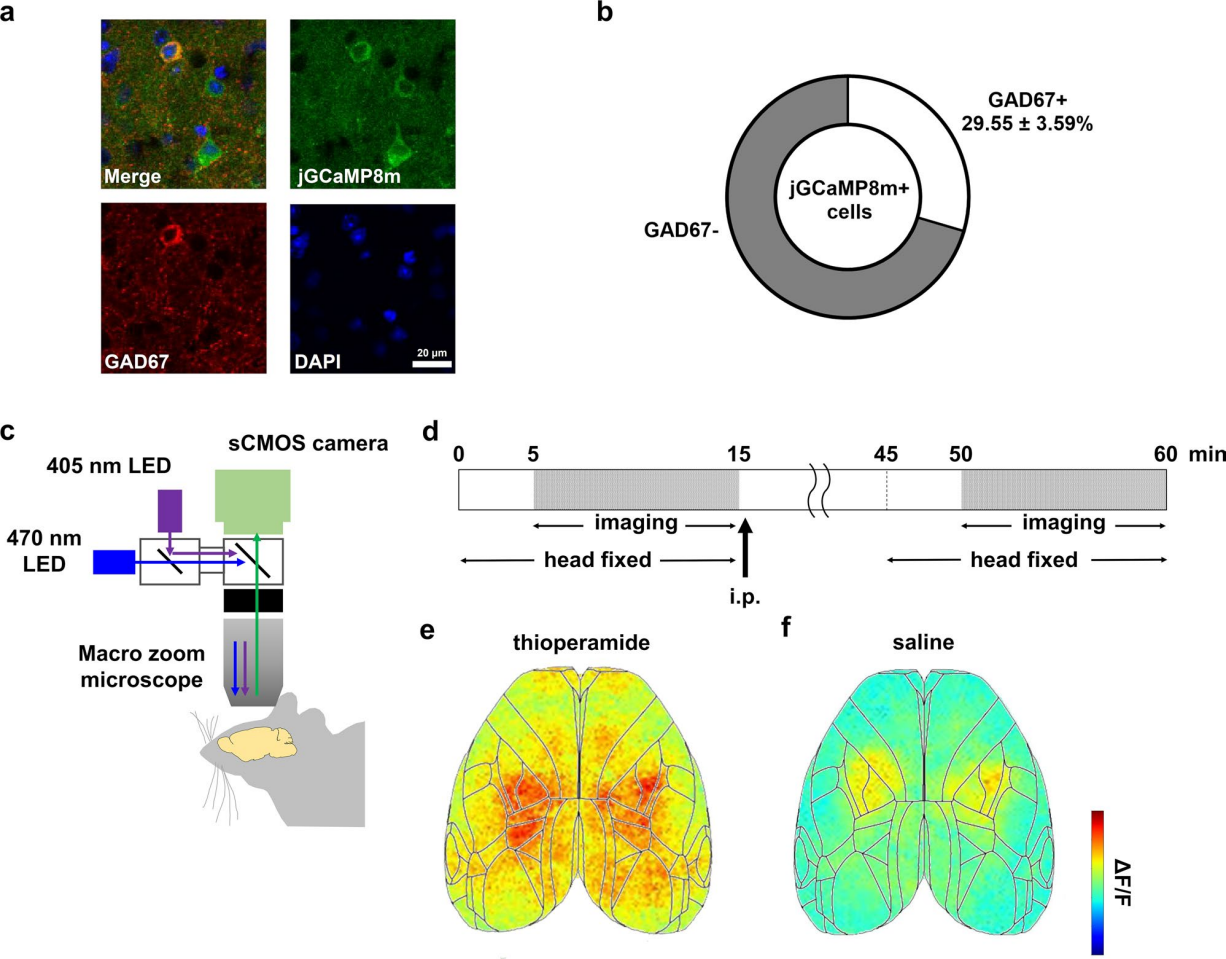
Cortex-wide Ca^2+^imaging, before and after drug administration. (**a**) Representative images showing GAD67 expression in jGCaMP8m-positive neurons. (**b**) The proportion of GAD67-positive neurons among jGCaMP8m-positive neurons (93.67 ± 8.45 neurons/mouse from 3 mice). (**c**) The head of the mouse was attached to a head fixation device, and the resting-state cortical activity was measured through the intact skull using a macro zoom microscope. (**d**) A 10-min imaging sequence was performed before and after drug administration over three consecutive days. (**e**) A representative image of cortical neuronal activity after thioperamide administration is shown. The acquired signals were corrected and z-score normalized. Lines represent the boundaries of cortical regions. (**f**) A representative image of cortical neuronal activity after saline administration is shown

**Fig. 2:**
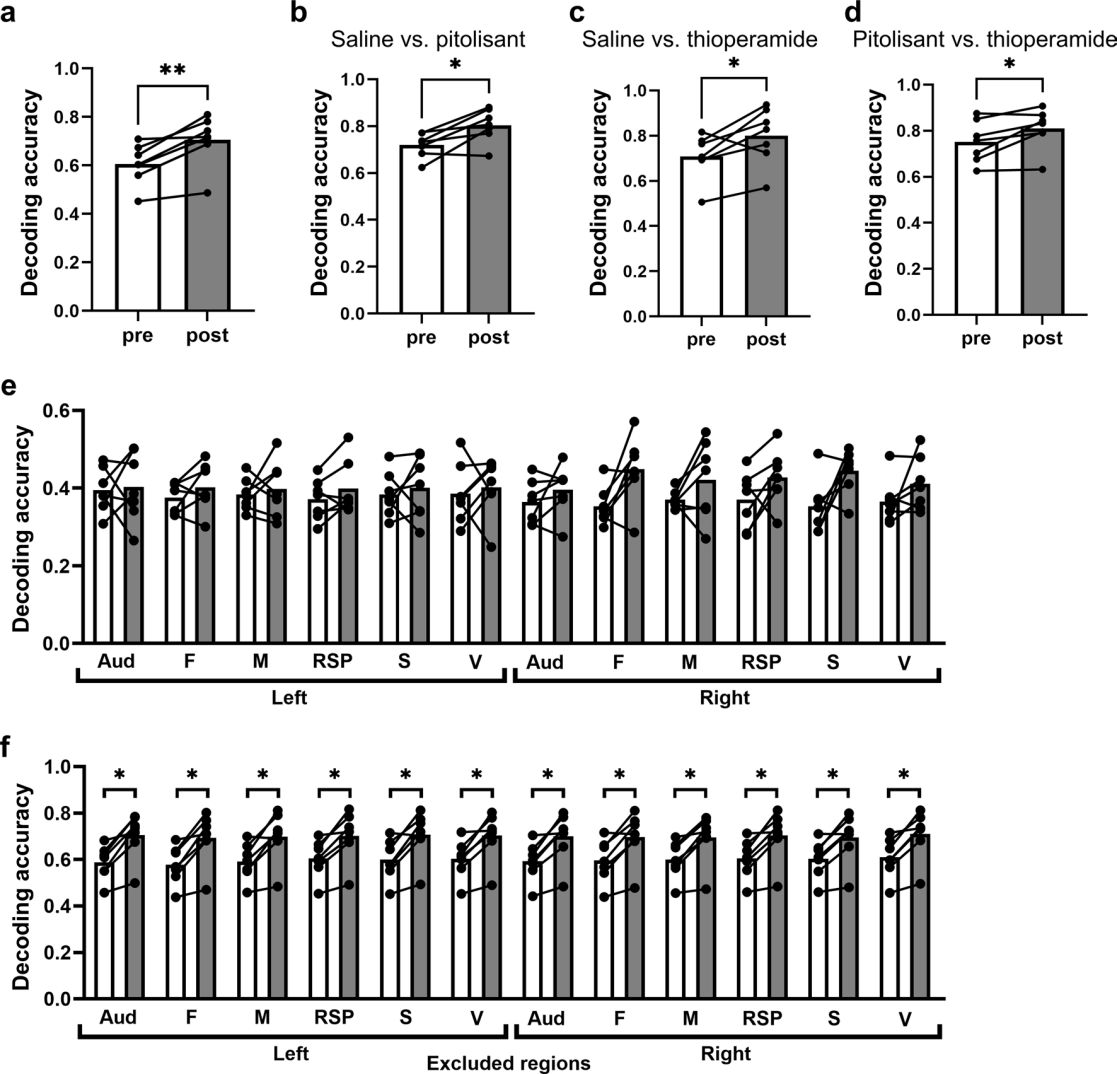
Identification of the drug administered from cortex-wide neuronal activity using a linear support vector machine. (**a**) The accuracy of the three-way decoder to discriminate between saline, thioperamide, and pitolisant administration is shown. Decoder performance improved when using neuronal activity data across the cerebral cortex after drug administration compared to data collected before drug administration (**p = 0.0035, paired t-test). (**b-d**) The accuracy of the two-way decoder to discriminate between saline and pitolisant (**b**), saline and thioperamide (**c**), and pitolisant and thioperamide (**d**) administration is shown. In all cases, decoder performance improved when using neuronal activity data after drug administration compared to data collected before drug administration (**b**, *p = 0.014; **c**, *p = 0.045; **d**, *p = 0.031; paired t-test). (**e**) The accuracy of the decoder based on activity data from a single region to discriminate saline, thioperamide, and pitolisant administration is shown, with no significant difference in accuracy when using data before or after drug administration for all cortical regions. (**f**) The accuracy of the decoder, based on entire cortical activity data excluding one region, to discriminate saline, thioperamide, and pitolisant administration is shown. Again, decoder performance improved when using post-administration activity data compared to pre-administration data across all cortical regions (*p < 0.05, paired t-test). Auditory cortex, Aud; prefrontal cortex, F; motor cortex, M; retrosplenial region, RSP; somatosensory cortex, S; and visual cortex, V

In vivo wide-field Ca^2+^ imaging was used to observe changes in cortex-wide neuronal activity following administration of histamine H_3_R inverse agonists/antagonists (Fig. 1). To achieve widespread expression of the fluorescent Ca^2+^ sensor jGCaMP8m throughout the cerebral cortex, the adeno-associated virus (AAV) PHP.eB-hSyn-jGCaMP8m was injected into the orbital venous plexus, which allowed us to measure changes in jGCaMP8m fluorescence in the dorsal cerebral cortex through the skull. We confirmed that 29.55 ± 3.59% of the jGCaMP8m + neurons were positive for GAD67 (Fig. 1a, b). After allowing at least 3 weeks for sufficient jGCaMP8m expression, cortex-wide calcium activity was recorded with the animals’ heads placed in a fixed frame and in the awake state, using a macro zoom microscope (Fig. 1c). Imaging sequences were performed on 3 consecutive days, at an interval of 24 h. For each imaging session, a 10-min sequence was obtained before and after administration of thioperamide, pitolisant (both H_3_R inverse agonists/antagonists drug), or saline (used as control reference) via intraperitoneal injection, with a 35-min interval between administration and image acquisition (Fig. 1d, e, f). To investigate whether histamine H_3_R inverse agonists/antagonists induced changes in neuronal activity across the cerebral cortex, we tested whether the type of administration (thioperamide, pitolisant, or saline) could be identified from the cortex-wide activity. We calculated the normalized (ΔF/F) activity for each cortical region and resampled the 10-min activity data sequences (a matrix of the 12 regions × 12,000 frames) into 10-s time bins (a matrix of the 12 regions × 60 bins) to focus on the effects on neural activity over a certain period of time, rather than momentary neural activity. We used the activity data with drug labels (60 each for thioperamide, pitolisant, and saline) to train three-way linear support vector machine decoders to distinguish the 3 different conditions (thioperamide, pitolisant, or saline) based on both pre- and post-administration activity data. The decoder distinguished the three drugs with an accuracy of 71% using post-administration activity data, which was higher than detection by chance (33%). Although the accuracy based on pre-administration data was 61%, exceeding the chance level due to increased distinguishability across observation periods (likely from baseline neural activity differences between experimental days), the accuracy based on post-administration remained higher than that based on pre-administration data (Fig. 2a). Furthermore, we created decoders to distinguish each pair of conditions (saline vs. pitolisant, Fig. 2b; saline vs. thioperamide, Fig. 2c; and pitolisant vs. thioperamide, Fig. 2d) based on both pre- and post-administration activity data. In all cases, post-administration accuracy was higher than pre-administration. Next, we analyzed the contribution of individual cortical regions. The decoder accuracy based on neuronal activity from each cortical region was comparable between pre- and post-administration data across all regions (Fig. 2e). However, decoder accuracy based on the set of neural activity of 11 regions, excluding one of the 12 defined cortical regions, was consistently greater for post- compared to pre-administration data (Fig. 2f). This analysis was conducted across 12 different patterns, each excluding a unique one of the 12 cortical regions. These results suggest that histamine H_3_R inverse agonists/antagonists alter the temporal activity patterns across the cerebral cortex.

### Reduction in the frequency of low-amplitude calcium events in the left somatosensory cortex

**Fig. 3:**
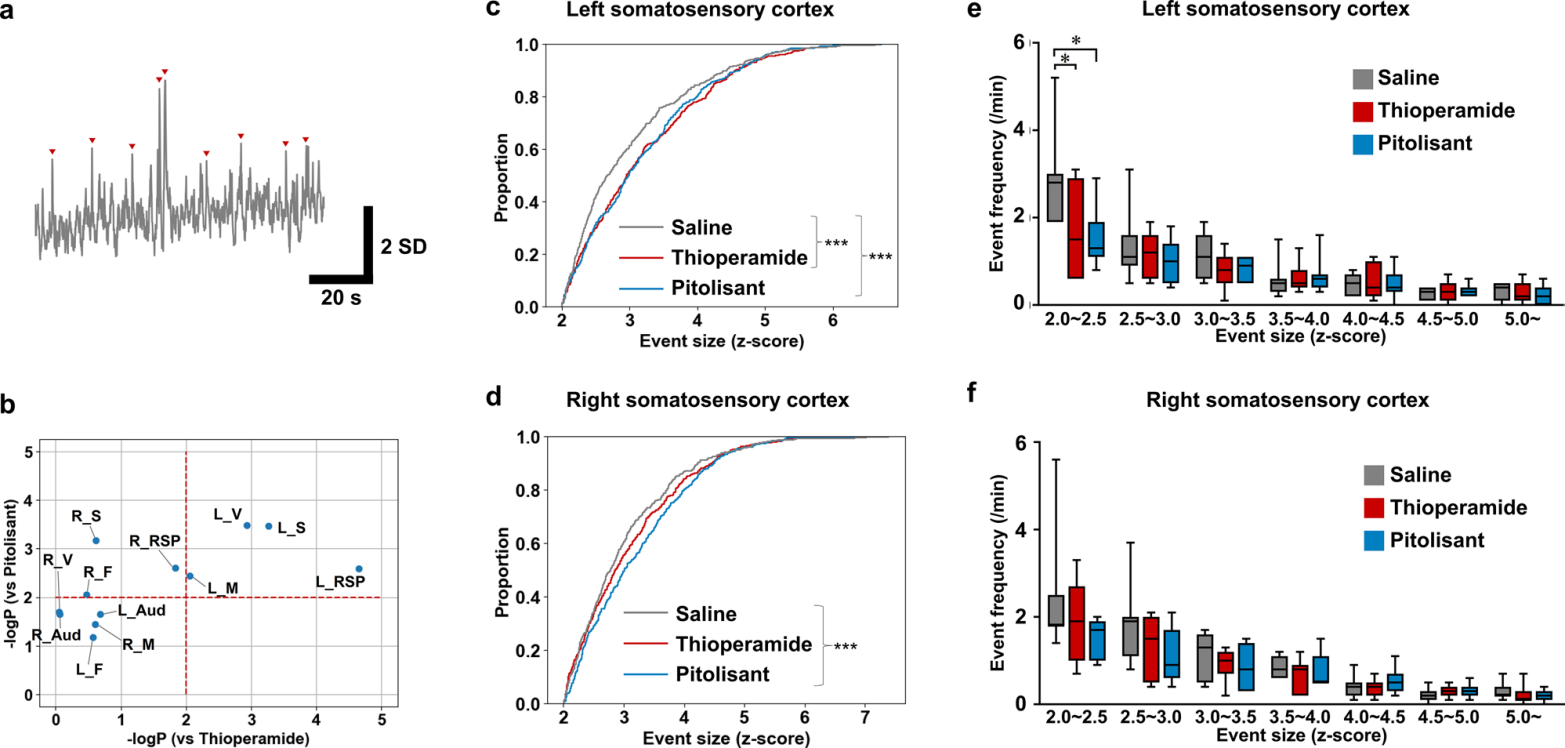
Histamine H _3_ receptor inverse agonists/antagonists drug administration decreases the frequency of small calcium events in the left somatosensory cortex. (**a**) A representative trace of z-scored GCaMP fluorescence in the left somatosensory cortex is shown. The red triangles indicate calcium events. (**b**) Changes in the distribution of calcium event amplitude in each cortical region are shown. The common logarithm of the p-values for comparisons between thioperamide and saline are plotted along the x-axis and for comparison of pitolisant and saline along the y-axis (red dotted lines indicate a p-value of 0.01, Kolmogorov–Smirnov test). (**c**) The cumulative relative frequency of calcium event amplitude in the left somatosensory cortex is shown. Thioperamide and pitolisant altered the distribution of calcium event amplitude (thioperamide vs. saline, ***p = 0.00054; pitolisant vs. saline, ***p = 0.00034; Kolmogorov–Smirnov test; saline, *n* = 494 events; thioperamide, *n* = 373 events; pitolisant, *n* = 362 events). (**d**) The cumulative relative frequency of calcium event amplitude in the right somatosensory cortex is shown. Pitolisant altered the distribution of calcium event amplitude (thioperamide vs. saline, *p* = 0.24; pitolisant vs. saline, ***p = 0.00067; Kolmogorov–Smirnov test; saline, *n* = 499 events; thioperamide, *n* = 395 events; pitolisant, *n* = 373 events). (**e**) Details of the distribution of calcium event intensity in the left somatosensory cortex are shown. Thioperamide and pitolisant reduced the frequency of low-amplitude calcium events (thioperamide vs. saline, *p = 0.032; pitolisant vs. saline, *p = 0.032; Dunn’s multiple comparisons test after Friedman test; *n* = 7 mice). (**f**) Details of the distribution of calcium event intensity in the right somatosensory cortex are shown. Data are represented as median and interquartile range (IQR). “R_” and “L_” indicate a right and left hemisphere region, respectively

The distribution of calcium event amplitudes (Fig. 3a), which characterize the neuronal activity in each cortical region, was compared for the 3 conditions (thioperamide, pitolisant, and saline). The H_3_R inverse agonists/antagonists didn’t alter the frequency of calcium events (Supplementary Fig.S1) but altered the distribution of calcium event amplitudes in multiple brain regions (Fig. 3b). Further analysis was conducted on the somatosensory and retrosplenial cortices, where changes in the distribution of calcium event amplitudes were observed bilaterally following the administration of H_3_R inverse agonists/antagonists. The left somatosensory cortex exhibited significant differences in calcium event amplitude distributions when either thioperamide or pitolisant was administered compared to saline. Based on these findings, we subsequently focused on the activity in the somatosensory cortex (Fig. 3c, d). In the left somatosensory cortex, a reduction in the frequency was observed for events in the smallest amplitude range (2.0 SD ~ 2.5 SD) with the use of both thioperamide and pitolisant (Fig. 3e). No changes in frequency were observed in the right somatosensory cortex with inverse agonists/antagonists administration (Fig. 3f). The same analysis on the retrosplenial cortex showed no significant differences between drug treatments (Supplementary Fig. S2). Moreover, we extended a similar analysis to all cortical regions; however, no regions exhibited consistent effects across thioperamide and pitolisant (Supplementary Fig. S2). Therefore, H_3_R inverse agonists/antagonists reduced the frequency of low-amplitude calcium events and increased the proportion of large-amplitude calcium events in the left somatosensory cortex.

### Enhancement of the connectivity in inter-regional cortical network in the right somatosensory cortex

**Fig. 4:**
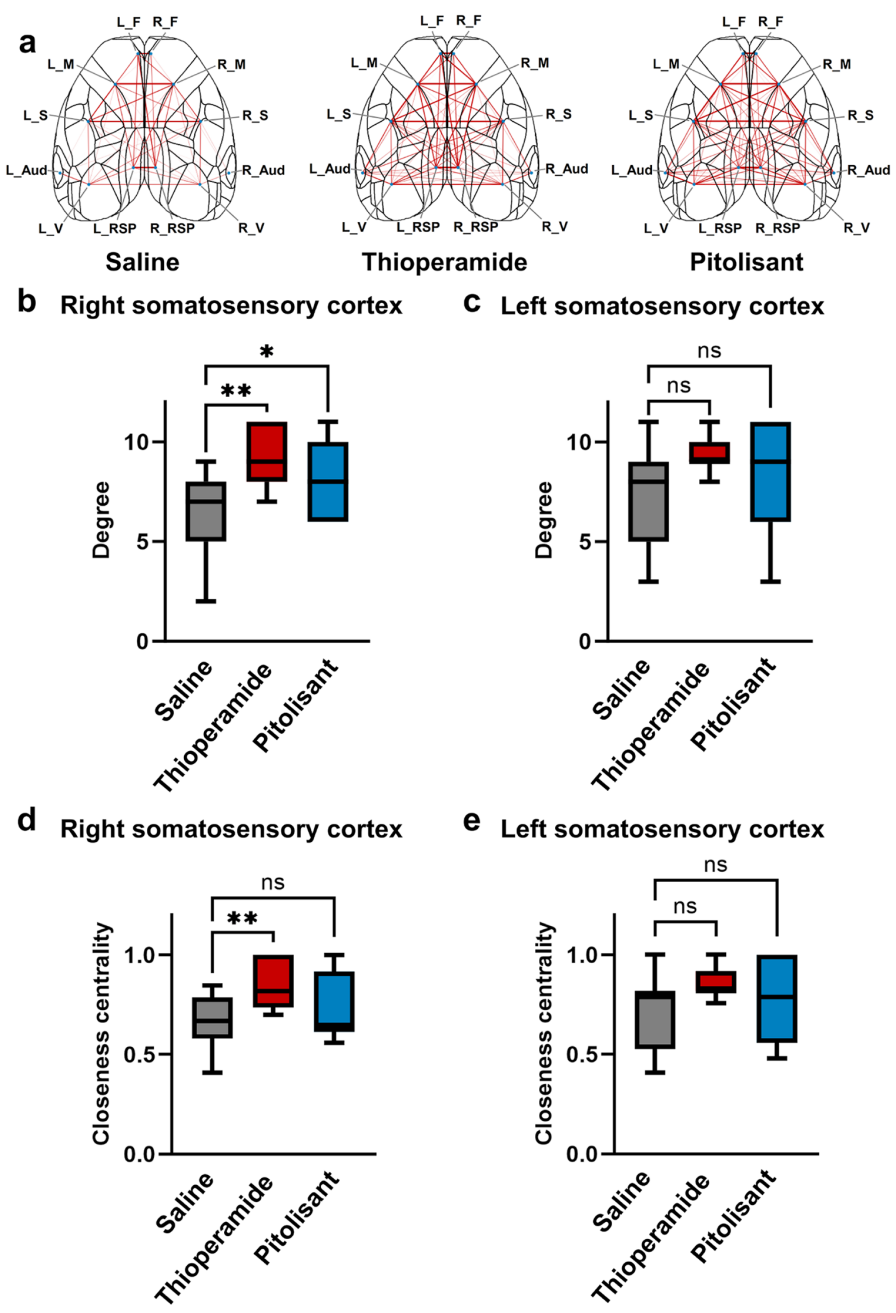
Histamine H _3_ receptor inverse agonists/antagonists drug administration enhances the importance of the right somatosensory cortex in the inter-regional cerebral cortex network. (**a**) A schematic diagram depicting the correlations between cerebral cortex regions after administration of saline, thioperamide, and pitolisant is shown. Results are based on data from a representative mouse, with edges indicating correlation coefficients ≥ 0.8 between connected regions. (**b**,** c**) Thioperamide and pitolisant increased ‘degree’ in the right (** b**) but not left (** c**) somatosensory cortex (**p = 0.0027, *p = 0.032, Dunn’s multiple comparisons test after Friedman test; *n* = 7 mice). (**d**,**e**) Thioperamide increased the ‘closeness centrality’ in the right (**d**) but not the left (**e**) somatosensory cortex (**p = 0.0042, Dunn’s multiple comparisons test after Friedman test; *n* = 7 mice). Data are shown as the median and interquartile range

Graph theory analysis was used to evaluate the effect of H_3_R inverse agonists/antagonists on the functional connectivity of cortical networks. The individual cortical regions were defined by nodes, with edges between regions defined by connections having a correlation coefficient ≥ 0.8 (Fig. 4a). The parametric variable of degree increased in the right somatosensory cortex with both thioperamide and pitolisant, compared to saline, administration (Fig. 4b), indicative of enhanced correlation of the right somatosensory cortex with more cortical regions after H_3_R inverse agonists/antagonists administration. The closeness centrality parameter in the right somatosensory cortex also increased with thioperamide, compared to saline, administration (Fig. 4d), suggesting that the right somatosensory cortex assumes a more central within the cortical network after thioperamide administration. By contrast, no differences in either the parameters of degree or closeness centrality were observed in the left somatosensory cortex (Fig. 4c, e). No differences in the clustering coefficient, local efficiency, or betweenness centrality were observed in either the right or left somatosensory cortices (Supplementary Fig. S3). Furthermore, there was no difference in any parameter in other regions (Supplementary Fig. S4, 5). Therefore, histamine H_3_R inverse agonists/antagonists increase the centrality of the right somatosensory cortex within the cerebral cortical network.

## Discussion

The key finding of our study was that histamine H_3_R inverse agonists/antagonists alter intra- and inter-regional cortical activity measured at resting state. These measured effects of inverse agonists/antagonists were confirmed by the increase in decoder accuracy to distinguish the condition (thioperamide, pitolisant, or saline) when using post- rather than pre-administration activity data. The effect of H_3_R inverse agonists/antagonists was not mediated by a change in neuronal activity in any single cortical region but rather due to activity changes across multiple regions and alterations in inter-regional connectivity. Specific drug-induced changes included a change in the amplitude distribution of calcium events across multiple regions, a reduction in low-amplitude calcium events in the left somatosensory cortex, and increases in the degree and closeness centrality parameters in the right somatosensory cortex. These increases suggest an enhanced role of the right somatosensory cortex as a central hub in the functional organization of the cortical network. The observed bilateral differences might indicate distinct mechanisms of effect H_3_R inverse agonists/antagonists for the right and left somatosensory cortex.

Neuronal synchronization plays crucial roles in information transfer, perception [34], memory formation [35], and memory retrieval [36]. We identified distributed effects of H_3_R inverse agonists/antagonists in modulating intra-regional neuronal synchronization across multiple cortical regions, indexed by alterations in the size of calcium events. Specifically, H_3_R inverse agonists/antagonists reduced the frequency of low-amplitude calcium events in the left somatosensory cortex without affecting high-amplitude events. The somatosensory cortex processes tactile and deep sensory information, and is implicated in cognitive functions, such as conditioned eyelid reflex and motor learning [37, 38]. By reducing small synchronized activity, histamine H_3_R inverse agonists/antagonists may influence information processing in these cognitive functions. We evaluated the impact of H_3_R inverse agonists/antagonists on inter-regional synchronization by graph theory measures based on inter-regional correlation coefficients. Changes in the centrality measures on graph theory analyses are closely related to cognitive function. Previous studies have shown that correlated activity between the secondary motor area and other cortical regions increases with the consolidation of motor learning [39]. This increase in correlation corresponds to an increase in the parametric measure of degree, which is related to memory consolidation. In addition, a study of cortical-wide activity during spontaneous movements revealed an increase in correlation coefficients and centrality measures in the somatosensory cortex during transitions between movement and rest [38], suggesting that centrality measures are associated with decision-making processes. In humans, changes in centrality measures have also been linked to cognitive function. For example, centrality decreases in the supplementary motor area, supramarginal gyrus, and anterior cingulate cortex during anesthesia and sleep [40]. Furthermore, age-related decreases in the degree parameter have been reported in the precuneus and posterior cingulate cortex [41], with a loss of inter-regional connections across the cortical network in patients with Alzheimer’s disease [42]. In this study, we demonstrated that histamine H_3_R inverse agonists/antagonists increased centrality measures in the right somatosensory cortex, which may enhance the function of this cortical area as a network hub, potentially leading to improvements in cognitive functions that depend on this region.

When considering the implications for our findings for future research, the limitations of our study need to be acknowledged. While this study revealed significant differences in the somatosensory cortex, further observations and analyses with cognitive tasks may uncover changes in other regions as well. While our previous research focused on cellular activity in the perirhinal cortex, this study investigated cortex-wide population activity, revealing that H_3_R inverse agonists/antagonists reduce the frequency of low-amplitude calcium events and enhance the connectivity in the inter-regional cortical network in the somatosensory cortex. Further investigation of cellular activity within the somatosensory cortex could potentially reveal synchronizations similar to those we previously observed at the cellular level in the perirhinal cortex [32]. The specific mechanisms underlying the effects of H_3_R inverse agonists/antagonists on the somatosensory cortices observed in this study remain unclear. These effects may be related to receptor expression patterns, pharmacological properties, or the involvement of specific signaling molecules. Additionally, we noted lateralized effects of histamine H_3_R inverse agonists/antagonists in the somatosensory cortex. Previous research has documented structural and functional differences between the left and right somatosensory cortices [43, 44]. Thus, the lateralization observed in our study may reflect these broader structural and functional asymmetries within the brain.

In conclusion, histamine H_3_R inverse agonists/antagonists alter the temporal activity patterns of various cortical regions, including the somatosensory cortex, and inter-regional connections during the resting state. Further investigations, combining memory tasks with our methods, and detailed analysis of the relevant cortical regions identified at the cellular level [45] may lead to a better understanding of how histamine H_3_R inverse agonists/antagonists improve cognitive functions.

## Methods

### Statement of ethics

All animal experiments were approved by the Institutional Animal Care and Use Committee of Nagoya City University (approval number: 22 − 018). The study method adhered to the Nagoya City University Guidelines for the Care and Use of Laboratory Animals and complied with relevant national guidelines: Guidelines for Proper Conduct of Animal Experiments (Science Council of Japan), Fundamental Guidelines for Proper Conduct of Animal Experiments and Related Activities in Academic Research Institutions (Ministry of Education, Culture, Sports, Science, and Technology, Notice No. 71 of 2006), and Standards for Breeding and Housing of and Pain Alleviation for Experimental Animals (Ministry of the Environment, Notice No. 88 of 2006).

### Animals model

Seven adult male C57BL/6 J mice (19–20 weeks old; Japan SLC, Hamamatsu, Japan) were used. Animals were housed individually in cages, at a constant ambient temperature (23 ± 1 °C), under a 12-h light/dark cycle (lights on from 7 a.m. to 7 p.m.), and with unrestricted access to food and water.

### Inverse agonists/antagonist drugs administered

Pitolisant hydrochloride (20 mg/kg, Sigma-Aldrich) and thioperamide maleate (20 mg/kg, Cayman) were dissolved in saline (0.9% NaCl) and administered to mice via intraperitoneal (i.p.) injection at a dose of 0.01 ml/g body weight. The control treatment consisted of an equal volume of saline. The pitolisant and thioperamide doses were selected based on previous studies [22, 32].

### Adeno-associated virus

AAV vectors were generated using the AAV Helper Free Expression System (Cell Biolabs, Inc., San Diego, CA) according to a previously published protocol [46]. Briefly, HEK293T cells were transfected with packaging plasmids (pUCmini-iCAP-PHP.eB [47] and pHelper) and a transfer plasmid (pGP-AAV-syn-jGCaMP8m-WPRE [48]) via the calcium phosphate method. The plasmids pUCmini-iCAP-PHP.eB and pGP-AAV-syn-jGCaMP8m-WPRE were gifts from Viviana Gradinaru (Addgene plasmid #103005; http://n2t.net/addgene:103005; RRID: Addgene_103005) and the GENIE Project (Addgene plasmid #162375; http://n2t.net/addgene:162375; RRID: Addgene_162375), respectively. AAV vector particles were extracted from the transfected cells as a crude cell extract and then purified through successive rounds of ultracentrifugation with cesium chloride. Following purification, the particles were dialyzed against phosphate-buffered saline (PBS) containing 0.001% Pluronic F-68 (Sigma-Aldrich, St. Louis, MO). Subsequently, the solution was concentrated using an Amicon 10 K MWCO filter (Merck Millipore, Darmstadt, Germany). The copy number of the viral genome (vg) was determined by real-time quantitative PCR.

### Surgical preparation of the head for imaging

To express jGCaMP8m throughout the cerebral cortex, mice were anesthetized using isoflurane and AAV PHP.eB-hSyn-jGCaMP8m (3.0 × 10^12^ vg/mL, 0.2 mL) was injected into the orbital venous plexus. At 1 week after the injection, surgery was performed to prepare the head for cortical activity imaging. Carprofen (5 mg/kg) and dexamethasone (0.2 mg/kg) were administered intraperitoneally, and the mice were then anesthetized with isoflurane (0.8–1.5%) before being placed in a stereotaxic frame (SR-6 M-HT, Narishige, Tokyo, Japan). After trimming scalp hair, lidocaine was topically applied for pain relief. The scalp was then removed and the connective tissue and blood on the skull were cleaned using a saline-soaked cotton swab and allowed to dry for 30 min. The exposed skull was then coated with a thin layer of cyanoacrylate adhesive (Aron Alpha, Tokyo, Japan) and allowed to dry for 30 min. A chamber frame (CF-10, Narishige, Tokyo, Japan) was then fixed using a self-curing adhesive resin cement (Super-Bond, SUN MEDICAL, Moriyama, Japan) along the lines connecting the left and right eyes and the line connecting the left and right ears. Eye shields were created using heat-shrink tubes and attached with instant adhesive to protect the eyes from excitation light during imaging, as well as to prevent body hair from entering the imaging area. To protect the coated skull, we applied a silicone impression material (DENT SILICONE-V, SHOFU, Kyoto, Japan) which was removed during imaging and reapplied afterward.

### In vivo wide-field calcium imaging

Wide-field calcium imaging was performed through the intact skull, using a macrozoom microscope (MVX10, Olympus, Tokyo, Japan), to simultaneously capture activity throughout the dorsal cortex. Ca^2+^-dependent jGCaMP8m signals were detected using blue light (470 nm LED light; Thorlabs, New Jersey, USA) reflected by a dichroic mirror onto the objective. Violet light (405 nm LED light, Thorlabs) was used for the reference images. Both fluorescence signals were captured using a high-speed CMOS camera (ORCA-Flash4.0; Hamamatsu Photonics, Hamamatsu, Japan), at a resolution of 512 × 512 pixels. The alternating illumination from the two LEDs was synchronized to the image capture using a custom Python script on a Raspberry Pi, with the signal and reference images at a rate of 11.7 fps.

Brain imaging was performed at > 2 weeks after the surgical preparation. Prior to imaging, the mice underwent habituation sessions over a 3-day period. During each habituation session, mice were attached to a custom-made head-fixation device for 10 min. These habituation sessions were conducted to ensure the mice maintained a resting state during the imaging sessions. Imaging sessions were performed over 3 consecutive days, as follows. The fixation device was attached to the head and, after a 5-min period of rest to allow the mice to maintain a resting state, the first 10-min imaging sequence was performed. After the imaging sequence, the fixation device was detached and an intraperitoneal injection of thioperamide, pitolisant, or saline was administered. The animal was then allowed to move freely in their cage for 30 min. Following this 30-min period, the head was attached to the fixation device and, again, after a 5-min period of rest, the second 10-min imaging sequence was performed. All animals received the thioperamide, pitolisant, or saline injections, one on each of the imaging days, with the order of drug administration randomly pre-determined for each animal.

### Immunohistochemistry

The mice were deeply anesthetized using an anesthetic mixture of medetomidine (0.75 mg/kg), midazolam (4.0 mg/kg), and butorphanol (5.0 mg/kg). They were then transcardially perfused with phosphate-buffered saline (PBS), followed by 4% paraformaldehyde (PFA) solution. The brains were post-fixed in 4% PFA overnight at 4°C, then cryoprotected in 15% and 30% sucrose solutions dissolved in PBS at 4°C for 48–72 h. Coronal slices, each 40 µm thick, were prepared using a cryostat (CM3050S, Leica). Tissue sections were washed three times with PBS for 5 min each, then blocked using B-PBS (0.3% Triton X-100, 10% Normal Goat Serum in PBS) for 1 h at room temperature (RT). These sections were incubated with a mouse primary antibody against glutamate decarboxylase 67 (GAD67; 1:500, MAB5406, Merck, NJ, USA) overnight at 4°C. Following antibody incubation, the sections were washed three times with PBST (0.3% Triton X-100 in PBS) for 5 min each, then incubated with Alexa Fluor 594-conjugated goat secondary antibody against mouse IgG (1:500, A11032, Thermo Fisher Scientific, MA, USA) and 4’,6-diamino-2-phenylidole (DAPI) (0.3 µg/ml, 4’,6-diamidino-2-phenylindole, 340–07971, Dojindo Laboratories, Kumamoto, Japan) for 2 h at RT. After two 5-min washes in PBST and two 5-min washes in PBS, the sections were mounted on glass slides with a mounting medium (20 mM Tris, 0.5% N-propyl gallate, 90% glycerol, pH 8.0). Imaging was performed using a laser-scanning confocal microscope (A1RS +, NIKON, Tokyo, Japan). The proportion of GAD67-positive cells within jGCaMP8m-positive cells in the somatosensory cortex was calculated.

### Signal processing and data analysis

The images were resampled to a resolution of 128 × 128 pixels. The images, acquired by alternating illumination with 470 nm and 405 nm light, were classified as ‘signal’ (470 nm excitation) and ‘reference’ (405 nm excitation), respectively. Each pixel was then normalized to the median fluorescence intensity over the entire imaging series (F) as ΔF/F. The 405 nm signal was then smoothed using a moving average window, 400-ms in width. The smoothed 405 nm ΔF/F signal for each pixel was regressed onto the 470 nm ΔF/F signal for the corresponding pixel, and the regression coefficients were then used to scale the 405 nm signal to match the 470 nm signal. The scaled 405 nm ΔF/F signal was then subtracted from the 470 nm ΔF/F signal to produce a normalized signal for each pixel.

To determine the cortical activation, images were projected onto the Allen Common Coordinate Framework for the mouse brain [49], aligning the exposed edges of the skull with the edges of the brain. The ΔF/F was then calculated for each of 58 subdivided cortical regions by averaging the ΔF/F of pixels within each region. The 58 subdivisions were then grouped into the following 12 larger brain regions: left and right somatosensory cortex (S; including SSp-bfd, SSp-ll, SSp-m, SSp-n, SSp-tr, SSp-ul, SSp-un and SSs); primary motor cortex (M; including MOp, MOs); visual cortex (V; including VISal, VISam, VISl, VISp, VISpl, VISpm, VISa, VISli, VISpor, VISrl); auditory cortex (Aud; including AUDd, AUDp, AUDpo, AUDv, TEa); retrosplenial cortex (RSP; including RSPagl, RSPd, RSPv); and prefrontal cortex (F; including ACAd, FRP, ORBm, PL). The acronyms for these regions are based on the Allen Common Coordinate Framework. The ΔF/F for each larger region was calculated as the average of its constituent subdivided regions. Whether drug administration could be identified from the cortex-wide activity was examined as follows. The 10-min normalized neuronal activity sequence, defined as the matrix of the 12 regions × 7,016 frames, was resampled into 10-s time bins, defined as the matrix of the 12 regions × 60 bins. This yielded a matrix of 12 regions × 180 bins, before and after the administration of each drug for each mouse. These activity data with drug labels − 60 labels each for pitolisant, thioperamide, and saline administration – were then used to train a linear support vector machine (SVM) decoder, using the Scikit-learn package. We used the SVM decoder with the following values: penalty value (C) = 30, gamma = scale, and kernel = RBF (radial base function). To determine the optimal C-value, we evaluated decoding accuracy for predicting the administered drugs (saline, thioperamide, or pitolisant) using a range of values: 1, 3, 10, 30, and 100. Among these, C-values of 10, 30, and 100 yielded similarly high decoding accuracies for post-administration data. Therefore, we selected a C-value of 30, as it is centrally positioned within this range, and used this setting for all subsequent analyses. The decoder performance was evaluated using cross-validation, with 80% of the data for training and 20% for testing. The decoder accuracy was computed as the average of 10,000 randomly selected datasets. To test whether the activity in each of the 12 large brain regions could predict drug administration, decoders were constructed based on data that included or excluded each cortical region and evaluated their performance.

To characterize the activity in each cortical region, calcium events were defined, as follows. The z-score for the F/F signal was calculated for each of the 12 defined cortical regions. To reduce noise effects, a median filter, with a kernel size of five, was applied. The activity was defined by data that exceeded 2 standard deviations (SD) of the surrounding signals, calculated using the scipy.signal.find_peaks function, with these occurrences designated as calcium events. Prominence, which measures how much a peak stands out owing to its height compared to the surrounding baseline, was considered the amplitude of a calcium event.

To investigate the characteristics of the cerebral cortex network, graph theory analysis was used. To calculate the interregional correlation coefficients, the z-scores for each region were averaged over one-second intervals. Nodes were defined as individual cortical regions, with the lines connecting the nodes shown when the absolute value of the correlation coefficient between the regions exceeds 0.8, with thicker lines indicating stronger correlation coefficients. The threshold for the correlation coefficient (*r* > 0.8) was determined based on a previous study [50]. This threshold selected the top 54.11 ± 19.28% of the connections out of all 66 connections between the 12 regions after administration of saline (mean ± SD, *n* = 7). Lastly, the ‘degree’, ‘clustering coefficient’, ‘local efficiency’, ‘betweenness centrality’, and ‘closeness centrality’ were calculated using the NetworkX package [51].

## Electronic supplementary material

Below is the link to the electronic supplementary material.

Supplementary Material 1

## Data Availability

All data generated during this study are included in this published article.

## Abbreviations

AAV: Adeno-associated virus
Aud: Auditory cortex
CMOS: Complementary metal-oxide-semiconductor
F: Prefrontal cortex
fMRI: Functional magnetic resonance imaging
GABA: γ-aminobutyric acid
H_3_R: Histamine H-3 receptors
i.p.: Intraperitoneal
LED: Light-emitting diode
M: Motor cortex
PRh: Perirhinal cortex
RSP: Retrosplenial cortex
S: Somatosensory cortex
SD: Standard deviation
SVM: Support vector machine
V: Visual cortex
