# Supplementary material for: Histamine H3 receptor inverse agonists/antagonists influence intra-regional cortical activity and inter-regional synchronization during resting state: an exploratory cortex-wide imaging study in mice
Source: Mol Brain. 2024 Nov 27;17:88. doi: 10.1186/s13041-024-01165-8 (PMC11603655; doi:10.1186/s13041-024-01165-8)
Supplement: Supplementary file 1 — Supplementary Material 1 [file 13041_2024_1165_MOESM1_ESM.docx]

**Supplementary Fig 1. Effects of histamine H_3_ receptor inverse agonists/antagonists on the frequency of calcium events across cortical regions.**

(a-l) The frequency of overall calcium events in the left somtatosensory (a), right somatosensory (b), left retrosplenial (c), right retrosplenial (d), left auditory (e), right auditory (f), left prefrontal (g), right prefrontal (h), left motor (i), right motor (j), left visual (k), right visual (l) cortex following drug administration is shown. Thioperamide and pitolisant didn’t alter the frequency of overall calcium events (Friedman test, n = 7 mice). Data represented as median with IQR.





**Supplementary Fig 2. Effects of histamine H_3_ receptor inverse agonists/antagonists on calcium events in the retrosplenial, auditory, prefrontal, motor, and visual cortex**

(a) Cumulative relative frequency of calcium event amplitude in the left retrosplenial cortex. Thioperamide and pitolisant altered the distribution of calcium event amplitude (thioperamide vs. saline p = 0.000022; pitolisant vs. saline p = 0.002599, **p < 0.01; ***p < 0.001, Kolmogorov–Smirnov test, saline n = 387; thioperamide n = 335; pitolisant n = 369). (b) Details of the distribution of calcium event intensity in the left retrosplenial cortex (Friedman test, n = 7 mice). (c) Cumulative relative frequency of calcium event amplitude in the right retrosplenial cortex. Pitolisant altered the distribution of calcium event amplitude (thioperamide vs. saline p = 0.014533; pitolisant vs. saline p = 0.002509, **p < 0.01, Kolmogorov–Smirnov test, saline n = 413; thioperamide n = 347; pitolisant n = 374). (d) Details of the distribution of calcium event intensity in the right retrosplenial cortex (Friedman test, n = 7 mice). (e) Cumulative relative frequency of calcium event amplitude in the left auditory cortex. Pitolisant altered the distribution of calcium event amplitude (pitolisant vs. saline p = 0.022463, Kolmogorov–Smirnov test, saline n = 425; thioperamide n = 395; pitolisant n = 358). (f) Details of the distribution of calcium event intensity in the left auditory cortex (Friedman test, n = 7 mice). (g) Cumulative relative frequency of calcium event amplitude in the right auditory cortex. Pitolisant altered the distribution of calcium event amplitude (pitolisant vs. saline p = 0.020024, Kolmogorov–Smirnov test, saline n = 429; thioperamide n = 378; pitolisant n = 382). (h) Details of the distribution of calcium event intensity in the right auditory cortex (Friedman test, n = 7 mice). (i) Cumulative relative frequency of calcium event amplitude in the left prefrontal cortex. Thioperamide and pitolisant didn’t alter the distribution of calcium event amplitude (Kolmogorov–Smirnov test, saline n = 249; thioperamide n = 278; pitolisant n = 266). (j) Details of the distribution of calcium event intensity in the left prefrontal cortex (Friedman test, n = 7 mice). (k) Cumulative relative frequency of calcium event amplitude in the right prefrontal cortex. Pitolisant altered the distribution of calcium event amplitude (pitolisant vs. saline p = 0.008772, **p < 0.01, Kolmogorov–Smirnov test, saline n = 252; thioperamide n = 272; pitolisant n = 234). (l) Details of the distribution of calcium event intensity in the right prefrontal cortex (Friedman test, n = 7 mice). (m) Cumulative relative frequency of calcium event amplitude in the left motor cortex. Thioperamide and pitolisant altered the distribution of calcium event amplitude (thioperamide vs. saline p = 0.008674; pitolisant vs. saline p = 0.003652, **p < 0.01, Kolmogorov–Smirnov test, saline n = 417; thioperamide n = 318; pitolisant n = 314). (n) Details of the distribution of calcium event intensity in the left motor cortex. Thioperamide reduced the frequency of low-amplitude calcium events (thioperamide vs. saline, *p = 0.0151; Dunn’s multiple comparisons test after Friedman test; n = 7 mice). (o) Cumulative relative frequency of calcium event amplitude in the right motor cortex. Pitolisant altered the distribution of calcium event amplitude (pitolisant vs. saline p = 0.036324, Kolmogorov–Smirnov test, saline n = 425; thioperamide n = 309; pitolisant n = 319). (p) Details of the distribution of calcium event intensity in the right motor cortex (Friedman test, n = 7 mice). (q) Cumulative relative frequency of calcium event amplitude in the left visual cortex. Thioperamide and pitolisant altered the distribution of calcium event amplitude (thioperamide vs. saline p = 0.001180; pitolisant vs. saline p = 0.000336, **p < 0.01; ***p < 0.001, Kolmogorov–Smirnov test, saline n = 425; thioperamide n = 381; pitolisant n = 376). (r) Details of the distribution of calcium event intensity in the left visual cortex (Friedman test, n = 7 mice). (s) Cumulative relative frequency of calcium event amplitude in the right visual cortex. Pitolisant altered the distribution of calcium event amplitude (pitolisant vs. saline p = 0.022170, Kolmogorov–Smirnov test, saline n = 418; thioperamide n = 385; pitolisant n = 377). (t) Details of the distribution of calcium event intensity in the right visual cortex (Friedman test, n = 7 mice). Data are represented as median with interquartile range (IQR).

**
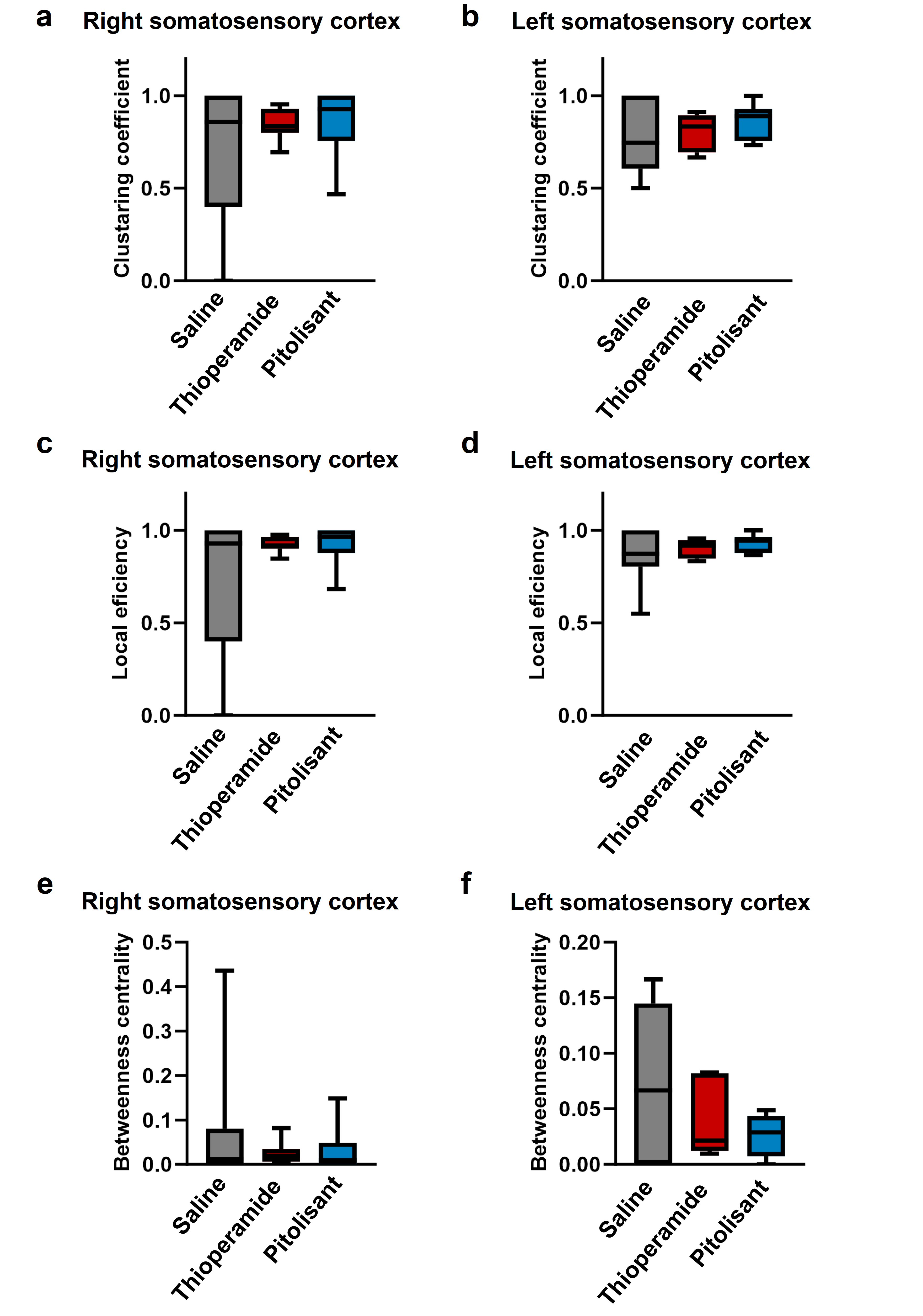
**

**Supplementary Fig 3. Effects of histamine H_3_ receptor inverse agonists/antagonists on measures in graph theory analysis**

(a, b) Thioperamide and pitolisant did not affect the ‘clustering coefficient’ in the right (a) and left (b) somatosensory cortex (Friedman test, n = 7 mice). (c, d) Thioperamide and pitolisant had no effect on ‘local efficiency’ in the right (c) and left (d) somatosensory cortex (Friedman test, n = 7 mice). (e, f) Thioperamide and pitolisant did not affect ‘betweenness centrality’ in the right (e) and left (f) somatosensory cortex (Friedman test, n = 7 mice). Data represented as median with IQR.

**

Supplementary Fig 4. Effects of histamine H_3_ receptor inverse agonists/antagonists on measures in graph theory analysis**

(a-e) Thioperamide and pitolisant did not affect ‘degree’ (a), ‘closeness centrality’ (b), ‘clustering coefficient’ (c), ‘local efficiency’ (d), and ‘betweenness centrality’ (e) in the right retrosplenial cortex (Friedman test, n = 7 mice). (f-j) Thioperamide and pitolisant did not affect ‘degree’ (f), ‘closeness centrality’ (g), ‘clustering coefficient’ (h), ‘local efficiency’ (i), and ‘betweenness centrality’ (j) in the right auditory cortex (Friedman test, n = 7 mice). (k-o) Thioperamide and pitolisant did not affect ‘degree’ (k), ‘closeness centrality’ (l), ‘clustering coefficient’ (m), ‘local efficiency’ (n), and ‘betweenness centrality’ (o) in the right prefrontal cortex (Friedman test, n = 7 mice). (p-t) Thioperamide and pitolisant did not affect ‘degree’ (p), ‘closeness centrality’ (q), ‘clustering coefficient’ (r), ‘local efficiency’ (s), and ‘betweenness centrality’ (t) in the right motor cortex (Friedman test, n = 7 mice). (u-y) Thioperamide and pitolisant did not affect ‘degree’ (u), ‘closeness centrality’ (v), ‘clustering coefficient’ (w), ‘local efficiency’ (x), and ‘betweenness centrality’ (y) in the right visual cortex (Friedman test, n = 7 mice). Data represented as median with IQR.



**Supplementary Fig 5. Effects of histamine H_3_ receptor inverse agonists/antagonists on measures in graph theory analysis**

(a-e) Thioperamide and pitolisant did not affect ‘degree’ (a), ‘closeness centrality’ (b), ‘clustering coefficient’ (c), ‘local efficiency’ (d), and ‘betweenness centrality’ (e) in the left retrosplenial cortex (Friedman test, n = 7 mice). (f-j) Thioperamide and pitolisant did not affect ‘degree’ (f), ‘closeness centrality’ (g), ‘clustering coefficient’ (h), ‘local efficiency’ (i), and ‘betweenness centrality’ (j) in the left auditory cortex (Friedman test, n = 7 mice). (k-o) Thioperamide and pitolisant did not affect ‘degree’ (k), ‘closeness centrality’ (l), ‘clustering coefficient’ (m), ‘local efficiency’ (n), and ‘betweenness centrality’ (o) in the left prefrontal cortex (Friedman test, n = 7 mice). (p-t) Thioperamide and pitolisant did not affect ‘degree’ (p), ‘closeness centrality’ (q), ‘clustering coefficient’ (r), ‘local efficiency’ (s), and ‘betweenness centrality’ (t) in the left motor cortex (Friedman test, n = 7 mice). (u-y) Thioperamide and pitolisant did not affect ‘degree’ (u), ‘closeness centrality’ (v), ‘clustering coefficient’ (w), ‘local efficiency’ (x), and ‘betweenness centrality’ (y) in the left visual cortex (Friedman test, n = 7 mice). Data represented as median with IQR.
